# 4024 Theory and Scale Development for Cancer-Related Self-Efficacy in Partners of Breast Cancer Survivors

**DOI:** 10.1017/cts.2020.439

**Published:** 2020-07-29

**Authors:** Andrea Cohee, Claire Draucker, Patrick Monahan, Victoria Champion

**Affiliations:** 1 Indiana University School of Nursing; 2 Indiana University School of Medicine

## Abstract

OBJECTIVES/GOALS: Specific aims are to: (Qualitative aim) Develop a new measure of cancer-related self-efficacy in partners (BCSES-P) and obtain feedback on the items (Quantitative) Evaluate the psychometric properties of the BCSES-P including: dimensionality, factor analysis, and construct validity assessing the relationships posited METHODS/STUDY POPULATION: 2-Phase Approach: 1) Item development and 1) Item testing Phase 1 Stage 1: Literature review to identify additional covariates Stage 2: Focus groups and individual interviews to determine partners’ needs Sample size: 20 partners (18 years of age or older, identifying as being in a committed relationship with a BCS) Design: cross-sectional, qualitative interviews Stage 3: Develop candidate items Stage 4: Cognitive interviews Stage 5: Finalize items with research team Phase 2 Preliminary psychometric testing Dimensionality Internal consistency reliability Construct validity Sample size: 150 partners Design: cross-sectional, online survey RESULTS/ANTICIPATED RESULTS: The BCSES-P will be unidimensional as assessed by exploratory factor analysis. The BCSES-P will demonstrate an internal consistency coefficient of 0.70 or above. Construct validity of the BCSES-P will be demonstrated by support of the following theoretical relationships: Cancer-related self-efficacy will be positively related to marital satisfaction and sexual functioning (social well-being) and the distal outcome, overall QoL. Cancer-related self-efficacy will be negatively related to fatigue (physical well-being), fear of recurrence, depression, and anxiety (psychological well-being). DISCUSSION/SIGNIFICANCE OF IMPACT: Findings will guide intervention development to improve partners’ quality of life The BCSES-P will be the first scale to measure partners’ cancer-related self-efficacy. This study will highlight a holistic approach to studying the long-term effects of breast cancer on partners.

